# Epigenetic tuning of brain signal entropy in emergent human social behavior

**DOI:** 10.1186/s12916-020-01683-x

**Published:** 2020-08-17

**Authors:** Meghan H. Puglia, Kathleen M. Krol, Manuela Missana, Cabell L. Williams, Travis S. Lillard, James P. Morris, Jessica J. Connelly, Tobias Grossmann

**Affiliations:** 1 Department of Psychology, University of Virginia, Charlottesville, VA 22904 USA; 2 Department of Neurology, University of Virginia, P.O. Box 800834, Charlottesville, VA 22908 USA; 3 Max Planck Institute for Human Cognitive and Brain Sciences, 04103 Leipzig, Germany; 4 Department of Early Child Development and Culture, Leipzig University, 04109 Leipzig, Germany

**Keywords:** Infant development, Social perception, OXTR epigenetics, EEG, Multiscale entropy

## Abstract

**Background:**

How the brain develops accurate models of the external world and generates appropriate behavioral responses is a vital question of widespread multidisciplinary interest. It is increasingly understood that brain signal variability—posited to enhance perception, facilitate flexible cognitive representations, and improve behavioral outcomes—plays an important role in neural and cognitive development. The ability to perceive, interpret, and respond to complex and dynamic social information is particularly critical for the development of adaptive learning and behavior. Social perception relies on oxytocin-regulated neural networks that emerge early in development.

**Methods:**

We tested the hypothesis that individual differences in the endogenous oxytocinergic system early in life may influence social behavioral outcomes by regulating variability in brain signaling during social perception. In study 1, 55 infants provided a saliva sample at 5 months of age for analysis of individual differences in the oxytocinergic system and underwent electroencephalography (EEG) while listening to human vocalizations at 8 months of age for the assessment of brain signal variability. Infant behavior was assessed via parental report. In study 2, 60 infants provided a saliva sample and underwent EEG while viewing faces and objects and listening to human speech and water sounds at 4 months of age. Infant behavior was assessed via parental report and eye tracking.

**Results:**

We show in two independent infant samples that increased brain signal entropy during social perception is in part explained by an epigenetic modification to the oxytocin receptor gene (*OXTR*) and accounts for significant individual differences in social behavior in the first year of life. These results are measure-, context-, and modality-specific: entropy, not standard deviation, links *OXTR* methylation and infant behavior; entropy evoked during social perception specifically explains social behavior only; and only entropy evoked during social auditory perception predicts infant vocalization behavior.

**Conclusions:**

Demonstrating these associations in infancy is critical for elucidating the neurobiological mechanisms accounting for individual differences in cognition and behavior relevant to neurodevelopmental disorders. Our results suggest that an epigenetic modification to the oxytocin receptor gene and brain signal entropy are useful indicators of social development and may hold potential diagnostic, therapeutic, and prognostic value.

## Background

Variability is a fundamental property of neural systems at multiple hierarchical levels, from the dynamics of ion channels to the convergence of multiple independent synaptic inputs [1, 2]. Recent work has capitalized on the inherently fluctuating nature of the brain to understand how variability in neural signals, which is often excluded from analysis as mere “noise,” may serve a valuable functional role [1–5]. Neural variability measured via electroencephalography (EEG) and functional magnetic resonance imaging (fMRI) shows strong links to both cognitive performance and cognitive development. Specifically, increased variability is associated with more accurate and stable perceptual, cognitive, and behavioral performance [6–11], and variability increases with development from infancy into adulthood [7–18]. These associations may occur because the addition of a moderate amount of random noise enhances and more accurately represents an underlying signal [2]. Such variability in brain activity also facilitates the exchange of information between neurons [19, 20], enhancing neural synchrony and promoting the formation of robust, adaptable networks that are not overly reliant on any particular node and display a greater dynamic range [2, 21, 22]. Together, these functions suggest that neural variability may act to appropriately weight incoming information such that important stimuli are maximally salient and enable the most flexible behavioral response.

For humans, social cues are highly important and particularly complex [23]. Social stimuli evoke unique neural and behavioral responses beginning in infancy [24] and require particular flexibility to generate appropriate behavioral responses across multiple contexts. Extensive work has demonstrated that oxytocin, a naturally occurring mammalian hormone, plays an important role in regulating social behavior across species [25], which has been posited to occur through a general effect on basic biological systems that facilitate the detection of and orientation to social information [26]. Oxytocin may facilitate such social signal detection by influencing neural variability in response to social stimuli; oxytocin directly regulates the firing rate of neurons in rodents, enhancing the signal-to-noise ratio, improving information transfer, and balancing neural inhibition and excitation [27–30]. When synaptic excitation and inhibition are properly balanced, signal variability is optimal and the neural system displays maximum information capacity, information transmission, and dynamic range [3, 19, 31–34].

We tested the hypothesis that individual differences in the endogenous oxytocinergic system early in life may influence infant social behavior by regulating variability in brain signaling during social perception in two independent studies. In study 1, infants provided a saliva sample at 5 months of age for analysis of individual differences in the oxytocinergic system and underwent EEG while listening to human vocalizations at 8 months of age for the assessment of brain signal variability. Infant behavior was assessed using the Revised Infant Behavior Questionnaire (IBQ-R), a widely used and validated measure of 14 domains of infant behavior and temperament based on parental report [35] that has been shown to correlate with overt behaviors [36–38] and to prospectively predict the development of subsequent traits [39–42]. In study 2, 4-month-old infants provided a saliva sample for analysis of individual differences in the oxytocinergic system and underwent EEG while viewing faces and objects and listening to human speech and water sounds. Infant behavior was assessed using the Short Revised Infant Behavior Questionnaire (IBQ-RS) [43] and a dynamic social interaction eye-tracking paradigm.

To assay individual differences in the human oxytocinergic system, we examined an epigenetic modification to the DNA of the oxytocin receptor gene, *OXTR* methylation, at cytosine-phosphate-guanine (CpG) site -934 (hg38_chr3:8,769,121-8,769,122). While currently available peripheral measures of the oxytocin peptide itself remain unreliable [44], this assay of its receptor is reliable, stable, and specific [45]. We have previously assayed methylation levels from all CpG sites within two *OXTR* CpG islands and shown that the level of DNA methylation at CpG site -934 is (1) significantly negatively associated with gene expression in human cortex [46], suggesting a regulatory role in gene transcription; (2) highly variable in the general population and associated with neural response during social perceptual tasks in neurotypical adults [47–49], suggesting it is a viable marker of individual differences in (endo) phenotypes; and (3) elevated in the brain and blood of both individuals with autism [46] and vole pups who experienced lower parental care early in life [50], suggesting this marker is indicative of individual developmental differences that are reflected in both causative (brain) and peripheral (blood) tissue.

We employed two methods for quantifying brain signal variability: standard deviation (SD) to measure overall distributional width (variance) of the signal and multiscale entropy (MSE) [51] to measure signal irregularity across temporal scales. Entropy tests for repeating patterns in a time series and assigns a high value to irregular signals and a low value to ordered, predictable signals. MSE is a popular technique in neuroscience because the brain is a complex system that operates on multiple time scales. In MSE, the time series is coarse grained (downsampled), and entropy is computed on the coarse-grained time series. When applied to brain signal data, MSE provides insight into the time-structure and linearity/nonlinearity of fluctuations in neural activity and network dynamics [52]. Entropy at short time scales captures both fast and slow oscillations and is thought to represent processing within local networks, whereas entropy at longer time scales captures slower oscillations and is thought to represent the integration of widely distributed cortical networks [52, 53].

Both SD [11, 54–56] and MSE [7, 8, 14, 15, 17, 57] have been positively associated with developmental and behavioral outcomes. However, no study to date has directly compared the explanatory power of these two variability measures, nor considered a role for oxytocinergic system function as an underlying molecular mechanism capable of contributing to brain signal variability during social perception in humans.

Early development is thought to constitute a period of increased sensitivity to the regulatory effects of epigenetic mechanisms [58], and infancy represents a particularly sensitive period in early development characterized by dramatic changes in brain structure and function [59]. Investigating specific epigene-brain-behavior associations early in development as done in the current studies can therefore be seen as a critical step in elucidating the mechanisms contributing to individual differences in social development.

## Methods

### Tissue comparison study

#### DNA collection and isolation

To determine the reliability of *OXTR* methylation values obtained from saliva, we first performed a tissue comparison study in which 207 healthy Caucasian adults (114 females) aged 16 to 81 years (*M* = 37.74, *SD* = 22.95) provided 5-mL passive drool in a Falcon 50 mL Conical Centrifuge Tube (Fisher Scientific, Hampton, NH) for assessment of saliva methylation and 8-mL blood in either mononuclear cell preparation tubes (BD Biosciences, Franklin Lanes, NJ) for assessment of peripheral blood mononuclear cell (PBMC) methylation (*n* = 142), or PAXgene Blood DNA Tubes (Qiagen, Valencia, CA) for assessment of whole blood methylation (*n* = 182). One hundred seventeen participants provided all three sample types. Saliva cells were pelleted in 20 mL 1x phosphate-buffered saline (Life Technologies, Carlsbad, CA) by centrifuging at 1800 rcf for 5 min. Pellets were then transferred into a microcentrifuge tube and frozen at − 20 °C prior to DNA extraction. We isolated DNA from saliva cells using reagents supplied in the QIAamp DNA Blood Mini Kit (Qiagen, Valencia, CA) following Qiagen’s Supplemental Protocol for Isolation of Genomic DNA from Saliva. We isolated DNA from peripheral blood mononuclear cells using reagents and protocol supplied in the Gentra Puregene Blood Kit (Qiagen, Valencia, CA). We isolated DNA from whole blood using reagents and protocol supplied in the PAXgene Blood DNA Kit (Qiagen, Valencia, CA).

#### Epigenetic analysis

Bisulfite conversion, polymerase chain reaction, and pyrosequencing were performed in triplicate exactly as detailed in Puglia et al. [48]. On average, replicates deviated from the mean ± 1.66%. PBMC methylation levels averaged 47.39% (*SD* = 6.42), whole blood methylation values averaged 46.61% (*SD* = 8.11), and saliva methylation values averaged 45.33% (*SD* = 6.46).

### Study 1

#### Participants

We report on a previously-acquired longitudinal dataset in which 81 healthy Caucasian infants (41 females) provided a saliva sample at 5 months of age (*M* = 149.93 days, *SD* = 14.29) and at 8 months of age (*M* = 247.54 days, *SD* = 5.87), underwent EEG, and received parent-reported behavioral ratings. All parents provided written informed consent prior to the study, approved by the Leipzig University Ethics Committee. Families were compensated with travel money, an infant t-shirt, a printed photo of the infant, and a toy for participation.

#### DNA collection and isolation

Passive drool was collected from infants at 5 months of age using CS-2 sponges and OG-250 kits (DNA Genotek, Ottawa, Canada) and was stored at room temperature until DNA isolation. We incubated collection kits at 50 °C for 1 h, then centrifuged for 10 min at 200 rcf to release all liquid from sponges. We isolated DNA from 500 μL of saliva using the manual purification protocol from DNA Genotek. DNA was stored in Hydration Solution (10 mM Tris, 1 mM EDTA, pH 7–8, Qiagen, Valencia, CA) and quantitated using nanodrop. One infant was excluded from the final analysis due to insufficient DNA.

#### Epigenetic analysis

Bisulfite conversion, polymerase chain reaction, and pyrosequencing were performed in triplicate exactly as in the tissue comparison study. On average, replicates deviated from the mean ± 1.83%. Infant saliva methylation levels averaged 40.34% (*SD* = 4.69).

#### EEG acquisition and preprocessing

Infants participated in an EEG paradigm in which they were presented with 4-s auditory clips of human vocalizations (infants crying, infants laughing, and neutral adult hummed speech). Stimuli were separated by a random inter-stimulus interval of 1000+ ms. Throughout the procedure, infants were seated on their parent’s lap in a dimly lit, sound-attenuated, and electrically shielded room. Infants were simultaneously presented with a non-social screensaver displaying animated bubbles to facilitate attention retention. Parents listened to classical music presented via headphones and were instructed not to talk to or interact with their infant during the course of the experiment. The EEG session ended when the infant became fussy or inattentive. EEG was acquired from 27 electrodes affixed to an elastic cap (EasyCap CmbH, Germany). Additional acquisition information is detailed in [60].

EEG preprocessing was completed using EEGLab, v14.1.1 [61], and progressed as detailed in prior infant [60] and MSE [8, 15] EEG studies. Data were bandpass filtered 0.3 to 20 Hz to remove slow drift and muscle artifacts [60], re-referenced to the average of all scalp electrodes [8, 15], and segmented into stimulus-evoked epochs 100 ms before stimulus onset to 1000 ms post stimulus onset with pre-baseline correction [60]. To assess spontaneous, ongoing variability, we randomly extracted 1100-ms epochs from the inter-stimulus interval that were not time-locked to the onset of a stimulus and did not overlap with stimulus-evoked epochs. Epochs contaminated with excessive amplitude standard deviations (> 100 μV in ocular channels, > 80 μV in scalp electrodes) within a 200-ms sliding window were discarded as artifacts [60]. Participants with at least 30 artifact-free auditory-evoked trials (10 from each social-auditory condition) and 30 artifact-free randomly sampled ongoing trials (*n* = 58) were retained in the analysis. This rejection rate (27.5%) can be compared to that reported in a meta-analysis of 149 infant EEG studies that found an average rejection rate of 49.2% [62].

We then completed an independent components analysis (ICA) on all remaining concatenated trials to remove components with clear ocular, muscular, or electrical artifacts identified via manual inspection of component topography, frequency, and time course [15]. On average, 4.43 (range 2 to 10) components were removed. The number of components removed did not correlate with MSE (*r* = .17, *p* = .171) or SD (*r* = −.08, *p* = .519) metrics, and removed components did not show significant event-related potential (ERP) effects (Table 1).

**Table 1:** ERP replication results

		ANOVA	Crying vs. neutral	Crying vs. laughing	Laughing vs. neutral
Processed data	N2	*F*(2,138) = 4.43, *p* = .014	*t*(69) = − 2.74, *p* = .008	*t*(69) = − 2.72, *p* = .008	*t*(69) = 0.05, *p* = .960
	P3	*F*(2,138) = 16.14, *p* < .001	*t*(69) = 0.85, *p* = .396	*t*(69) = − 4.93, *p* < .001	*t*(69) = 4.66, *p* < .001
	LP	*F*(2,138) = 4.55, *p* = .012	*t*(69) = 2.13, *p* = .037	*t*(69) = − 0.95, *p* = .346	*t*(69) = 2.88, *p* = .005
Rejected ICA components	N2	*F*(2,138) = 0.91, *p* = .406	*t*(69) = 1.28, *p* = .206	*t*(69) = 0.7, *p* = .485	*t*(69) = 0.69, *p* = .495
	P3	*F*(2,138) = 2.21, *p* = .113	*t*(69) = 1.9, *p* = .062	*t*(69) = 0.98, *p* = .329	*t*(69) = 1.3, *p* = .197
	LP	*F*(2,138) = 0.72, *p* = .489	*t*(69) = 0.92, *p* = .360	*t*(69) = 1.02, *p* = .310	*t*(69) = 0.22, *p* = .826

Processed data: ANOVA results replicating ERP N2, P3, and LP effects reported in the original study by Missana et al. [60] using the novel preprocessing steps taken in this secondary data analysis in study 1. Rejected ICA components: We find no significant differences across conditions in the ICA components rejected during preprocessing, suggesting these data were correctly rejected as artifacts

Because the number of data points included in MSE calculation can influence reliability of the estimates [63], we selected the 30 auditory-evoked (10 from each social-auditory condition) and 30 spontaneous-ongoing trials with total global field power (GFP) [8] closest to the median GFP for each participant for inclusion in downstream analyses (16,470 data points per condition). This selection criterion identifies the most representative trials for each individual and minimizes the influence of trials potentially contaminated with high residual amplitude artifacts [8]. To ensure that the current procedures did not obscure or eliminate relevant evoked response, we reproduced all results from Missana et al. [60] using these data re-referenced to the average of the mastoids (Table 1).

#### Brain signal variability analyses

After preprocessing, we subjected these thirty 1100-ms auditory-evoked epochs and thirty 1100-ms spontaneous-ongoing epochs to brain signal variability analyses. Brain signal variability can be quantified in many ways [3]. Here we consider two of the most commonly applied measures of brain signal variability—SD, a measure of signal variance, and MSE, a measure of temporal irregularity.

##### Multiscale entropy

MSE [51] computation involves (1) coarse graining the time series to scale *s* by averaging together *s* successive, non-overlapping points, and (2) computing sample entropy [64]. Sample entropy is a measure of signal irregularity in which two patterns of *m* length are considered indistinguishable if each point *k* is within *k* ± *r*. Then, the natural log of the ratio of the count of *m* patterns to the count of *m* + 1 patterns is computed. Higher sample entropy values therefore indicate higher irregularity in the data because patterns of length *m* + 1 reoccur less often than patterns of length *m*.

In Costa’s original MSE algorithm [51], *r* is calculated as a percentage of SD of the original time series and remains constant across all scales. However, this method conflates entropy with variance [63, 65]. We therefore used a modified algorithm [65] that recalculates *r* at each scale as a percentage of SD of the coarse-grained time series. We computed MSE on the residuals of the EEG signal (i.e., after subtracting the within-person average response across the 30 trials within each condition) for the 30 auditory-evoked and 30 spontaneous-ongoing trials for each scalp electrode using the algorithm described in Grandy et al. [63] for estimating MSE across discontinuous segments, modified to recalculate *r* for each scale. Trials from all social auditory conditions were combined in the auditory-evoked analysis to provide sufficient data points for accurate MSE estimation [63]. Parameter values were set to pattern length *m*  =  2 and similarity criterion *r*  =  .5, the most commonly used and recommended values ([52, 63], see also [63] which found no substantial effect on the accuracy and precision of MSE estimates across multiple *m* and *r* parameter settings).

**Fig. 1:**
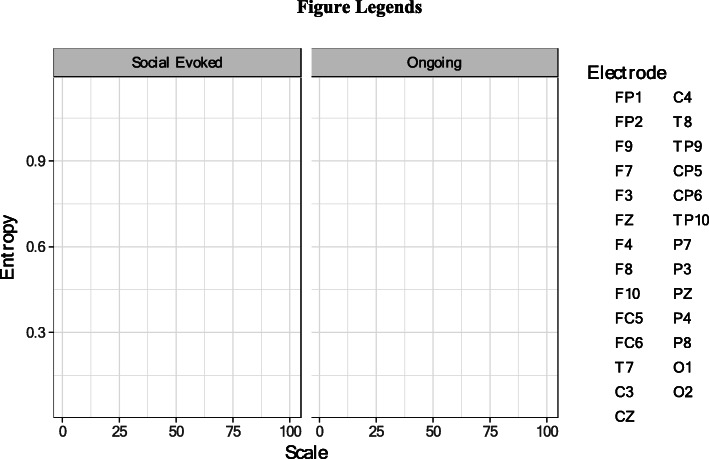
Group average multiscale entropy curves. Study 1 average multiscale entropy curves from for scales 1 to 100 (500 to 5 Hz) are plotted for the social evoked (left) and ongoing (right) EEG signal for each electrode (*n* = 55)

Here, we focused our primary analyses on the area under the multiscale entropy curve (MSE_*AUC*_) for each of the 27 electrodes for scales 1 to 100 (corresponding to 500 to 5 Hz) to obtain a comprehensive picture of the temperodynamic structure of our data. Average MSE curves are plotted in Fig. 1, and average area under the curve values are listed in Table 2. We also considered entropy at scales 1 (MSE_*1*_, 500 Hz), 50 (MSE_*50*_, 10 Hz), and 100 (MSE_*100*_, 5 Hz) to assess the impact of specific time scales on our models in secondary analyses. We found scales 37 to 60 (8 to 13 Hz) show the highest correlation with MSE_*AUC*_ across all electrodes, suggesting these scales, in particular, may drive our results with MSE_*AUC*_.

**Table 2:** Average area under the multiscale entropy curve values

Electrode	Evoked	Ongoing
FP1	89.61 (1.58)	89.20 (1.59)
FP2	89.46 (1.56)	89.92 (1.46)
F9	85.26 (1.55)	84.71 (1.69)
F7	89.04 (1.66)	89.49 (1.59)
F3	89.27 (1.72)	89.43 (1.70)
FZ	90.62 (1.38)	90.22 (1.48)
F4	91.43 (1.51)	92.38 (1.66)
F8	89.71 (1.55)	89.63 (1.53)
F10	86.56 (1.45)	84.51 (1.51)
FC5	91.11 (1.51)	92.11 (1.75)
FC6	92.39 (1.83)	91.82 (1.73)
T7	87.66 (1.83)	88.25 (1.85)
C3	86.21 (1.66)	86.31 (1.68)
CZ	89.00 (1.54)	88.87 (1.64)
C4	88.88 (1.79)	89.02 (1.70)
T8	91.71 (1.87)	89.52 (1.91)
TP9	90.44 (1.56)	93.29 (1.57)
CP5	94.04 (1.24)	93.00 (1.28)
CP6	93.38 (1.41)	93.48 (1.37)
TP10	88.46 (1.63)	92.67 (1.45)
P7	94.45 (1.51)	95.20 (1.50)
P3	90.18 (1.52)	87.76 (1.44)
PZ	88.10 (1.59)	87.66 (1.74)
P4	90.47 (1.50)	88.41 (1.65)
P8	95.20 (1.26)	95.77 (1.22)
O1	91.69 (1.27)	92.36 (0.99)
O2	92.23 (1.14)	92.14 (1.15)

Mean and (standard error of the mean) area under the multiscale entropy curve values for each electrode and condition in study 1

##### Standard deviation analysis

To capture moment-to-moment variance in the EEG signal, we calculated SD on the residuals of the EEG signal (i.e., after subtracting the within-person average ERP) for the 30 auditory-evoked and 30 spontaneous-ongoing trials for each of the 27 electrodes using two methods commonly reported in the literature. Specifically, SD can be calculated across the continuous time series as a measure of distributional width (SD_*CONT*_, the focus of our primary analyses) (e.g., [66]), or across trials as a measure of the trial-by-trial reliability of the evoked response (SD_*TXT*_) (e.g., [54]). These computation methods yield highly correlated values (all *r*s ≥ .99). To equalize data volume and computation across our brain signal variability metrics, we also consider the area under the course-grained SD curve (SD_*AUC*_), and SD at scales 1 (SD_*1*_, equivalent to SD_*CONT*_), 50 (SD_*50*_), and 100 (SD_*100*_) in secondary analyses.

**Fig. 2:**
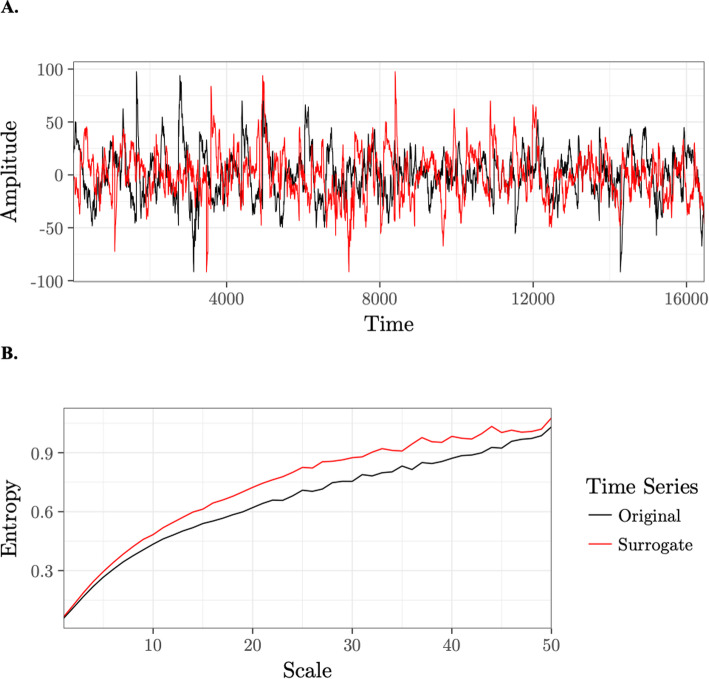
An illustration of the temporal dependency of multiscale entropy. **a** We created a surrogate time series (red) by randomly shuffling segments of the original time series (black) consisting of actual EEG data from one trial. The standard deviations of the original and surrogate time series are equivalent, 22.63. **b** We find higher entropy for the surrogate time series (red) than the original time series (black) across time scales because the scrambling procedure introduced greater temporal irregularity into the surrogate time series

Finally, because entropy explicitly incorporates signal SD when defining the similarity criterion parameter *r*, *r* is larger for a signal with greater SD, meaning the entropy algorithm is more likely to identify matches, resulting in a lower entropy value [67]. However, only MSE is sensitive to temporal dependencies in a signal (Fig. 2). To understanding the unique contribution of entropy and variance of a signal on our models, we orthogonalized MSE and SD estimates by regressing SD from MSE scale-wise for each participant, electrode, and condition prior to computing the area under the curve (AUC) in secondary analyses (MSE_*SDRes*_).

#### Infant behavior

Infant behavior was assessed via parental report with the IBQ-R [35], a 191-item measure organized into 14 subscales that is designed to assess infant behavior and temperament from 3 to 12 months of age. Parents rate how frequently their infant displayed specific behaviors on a scale ranging from 1 (never) to 7 (always).

To separately consider social and non-social aspects of infant behavior, we created new Social and Non-Social IBQ-R constructs. We first manually identified unambiguously social and non-social items by subscale. Then, we conducted an item analysis to ensure that included items constituted a valid measure of each construct using Cronbach’s *α*, a measure of internal consistency that determines the correlation among related items, implemented in the psych package in R [68, 69]. Specifically, we removed items until Cronbach’s *α* > .70 [70] for all Social and Non-Social subscales or until the removal of additional items did not improve α. Subscales that did not achieve *α* > .60 were not considered further. Models 2 and 3 were run with Social and Non-Social constructs both before and after item removal. Results did not appreciably change after item removal; results are presented after item removal. Finally, we tested for construct validity to confirm that the Social and Non-Social subscales were identified as unique, distinguishable constructs in the model by confirming that the loadings for each subscale exceeded .5 [71] for its own construct (Social IBQ-R *M* = .55, Non-Social IBQ-R: *M* = .68) and that the cross-loadings for each subscale did not exceed .5 onto the other construct (Social IBQ-R: *M* = .02, Non-Social IBQ-R: *M* = − .01). Individual items included in the final Social and Non-Social IBQ-R constructs are listed in Table 3.

**Table 3:** Social and Non-Social IBQ constructs

Construct	Subscore	***α***	Items
Social IBQ-R	Approach	0.59	172, 173
	Cuddliness	0.88	5, 6, 7, 105, 106, 107, 108, 123, 124, 125, 126, 127, 128, 129, 130, 131, 132
	Duration of orientation	0.68	55, 101
	Fear	0.9	90, 150, 151, 152, 153, 154, 155, 156, 161, 162, 163, 164
	High pleasure	0.72	58, 59, 60, 61, 65, 66, 67, 77, 78, 79, 80, 81, 165
	Soothability	0.71	174, 176, 177, 178, 179, 189, 190, 191
	Vocalization	0.73	8, 9, 10, 35, 42, 45, 52, 102, 103, 146, 147, 148
Non-Social IBQ-R	Approach	0.7	85, 86, 87, 88, 97, 98, 160
	Duration of orientation	0.7	46, 47, 48, 49, 50, 51, 54, 91, 92
	Fear	0.78	157, 158
	Perceptual sensitivity	0.78	4, 83, 95, 96, 133, 134, 135, 136, 137, 138, 139
	High pleasure	0.78	82, 62, 63, 64, 68, 69, 70, 71, 72, 73, 74
	Soothability	0.68	183, 184, 186, 187

Individual items included in the Social and Non-Social Revised Infant Behavior Questionnaire (IBQ-R) constructs after item analysis. *Abbreviations*: *α* Cronbach’s alpha

#### Experimental design and statistical analysis

We employed a multivariate, prediction-based approach to develop models of our hypothesized epigene-brain-behavior associations with partial least squares path modeling (PLS-PM). PLS-PM is a statistical method for studying complex multivariate associations among measured and latent variables [71]. A path model consists of an inner model, in which associations (path coefficients) are calculated between constructs (latent variables, a predictor points to a criterion), and an outer model, in which associations (loadings) are calculated between the original measured variables (indicators) and their latent variables. Factor scores are calculated for each observation as a weighted average of the measured variables.

Particularly given the novelty of the present research, PLS is better suited than other multivariate techniques like covariance-based structural equation modeling (CBSEM) because PLS is considered optimal for exploratory, prediction-based research where theory is less developed [71, 72]. Complex models with many observed variables and relationships can be estimated with smaller sample sizes with PLS than required by CBSEM [72]. Unlike CBSEM, PLS is a nonparametric technique which makes no assumptions about the normality of the distribution of the data. Furthermore, PLS is well suited to the highly dimensional, highly correlated nature of neuroimaging data (i.e., among many electrodes, voxels) [73]. For these reasons, PLS has become a popular and commonly used modeling technique within neuroimaging [3, 7–9, 11, 15, 66, 74].

##### Model specification and assessment

Traditionally, it is assumed that there is a system of linear associations between latent variables. However, to account for potential curvilinear [75–77] associations among our epigene-brain-behavior variables, we estimated all models using WarpPLS v6.0, the only software currently available to explicitly identify nonlinear functions connecting pairs of latent variables [78].

For all models, we estimated a reflexive outer model for all constructs using the PLS regression algorithm with WarpPLS. Methylation values generated in each of the 3 replicate analyses served as indicators for the epigene (*OXTR* methylation) latent variable, data from each of the 27 electrodes served as indicators for the brain (MSE, SD) latent variables, and scores from each subscale served as indicators for the behavior (IBQ-R, 14; Social IBQ-R, 7; Non-social IBQ-R, 6) latent variables. Inner model path coefficients were estimated using the Warp2 algorithm, which tests for second-order polynomial associations among latent variables through best-fitting nonlinear u-curve or j-curve functions (e.g., logarithmic, hyperbolic decay, exponential decay, exponential, quadratic relationships, etc.) that minimize sums of squared residuals on a bivariate basis. These functions are identified through the following equation, where F1(LVp1) and F2(LVp2) are nonlinear functions that relate blocks of predictor latent variables (LVp1, LVp2) to a criterion latent variable (LVc), p1 and p2 are the path coefficients, and E is the error term [78, 79]:

$$ LVc=p1\ast F1(LVp1)+p2\ast F2(LVp2)+E $$

If the algorithm determines that a curvilinear relationship best fits the distribution of points associated with a pair of latent variables, WarpPLS first performs nonlinear transformations on the factor scores and then calculates the path coefficient between latent variables, assigning the sign of the coefficient to be the sign of the correlation between latent variables prior to the nonlinear transformation [78, 79]. If the algorithm determines that a linear or quasilinear relationship best fits the distribution of points associated with a pair of latent variables, WarpPLS calculates the path coefficient between latent variables without performing nonlinear transformations [78, 79]. The best-fitting curves are representations of the identified functions. However, when high measurement error exists in the dataset, there is increased likelihood that the identified functions are distorted by this error and do not represent the true underlying functions. To determine whether the nonlinear functions discovered by WarpPLS represent good approximations of the true underlying functions, one can divide the dataset into quantiles and plot the mean value of each criterion latent variable for each quantile. If visual inspection of these quantile plots mimics the shape of the best-fitting curve, one can conclude that the identified function is a good representation of the true underlying function [78], p. 98. Quartile plots for all significant effects for each model can be viewed in Additional file 1.

##### Outliers

After initial model fitting, values were considered outliers if the factor score fell more than 3 median absolute deviations from the median. We selected this relatively conservative criterion to balance outlier detection with subject retention. We first determined whether these outliers were driven by a single indicator within blocks. Methylation values for two subjects were identified as outliers in single replicates. These outlier replicates were removed and imputed with the mean of the other two replicate values for these subjects. One infant was identified as an outlier across all three replicates; one infant was identified as an outlier in both MSE and SD factor scores; one infant was identified as an outlier in the behavioral factor scores but was not an outlier in any single behavioral indicator. These three participants were removed, and models were re-estimated. Results did not appreciably change with or without outliers; we therefore conservatively report on models excluding outliers (*n* = 55, 29 females).

After removal of outliers, we identified indicators with negative loadings and reverse-coded these items. For models with original IBQ-R subscores, these items included Activity Level, Distress to Limitations, Fear, Perceptual Sensitivity, and Sadness. For models with Social/Non-Social IBQ-R subscores, these items included Social Fear and Non-Social Fear.

Next, we checked for convergent and discriminant validity by identifying and removing any items that did not significantly load onto its construct or that loaded higher onto another construct for each model. These included Duration of Orientation, Perceptual Sensitivity, Vocal Reactivity, Social Duration of Orientation, Non-Social Fear, Non-Social Duration of Orientation, and Non-Social High Pleasure. Removing these items did not appreciably change results; results are presented with these items removed.

Finally, we determined that the square root of the average variance extracted (AVE) was greater than the correlations between constructs. We report construct internal consistency and reliability as indexed through the composite reliability coefficient (recommended value > .60, [80]), and explanatory power through *R*^2^ values in Table 4 for each model. For all significant effects, we report path coefficients (*β*), standard errors for path coefficients (*SE*), *p* values (*p*) estimated with delete-1 jackknifing, and effect sizes analogous to Cohen’s *f*^2^ coefficient [81]. WarpPLS calculates effect sizes as the absolute values of the individual contributions of the corresponding predictor latent variables to the *R*^2^ coefficients of the criterion latent variable in each latent variable block [82]. The reported *f*^2^ statistics can be interpreted as recommended by Cohen [81]—.02, .15, and .35 indicate small, medium, and large effect sizes, respectively.

**Table 4:** Model quality indices

**Composite reliability coefficients**						
**Model**	*OXTR*m	MSE_AUC_	SD_CONT_	IBQ-R	Social IBQ-R	Non-Social IBQ-R
1	0.87	0.96	0.96	0.82	–	–
2	0.87	0.96	0.96	–	0.73	0.78
3	0.87	0.97	0.95	–	0.73	0.74
**Study 2**	*OXTR*m	Auditory MSE_AUC_	Visual MSE_AUC_	Verbal behavior	Visual behavior	
	0.82	0.75	0.79	0.84	0.90	
***R***^**2**^**coefficients**						
**Model**	*OXTR*m	MSE_AUC_	SD_CONT_	IBQ-R	Social IBQ-R	Non-Social IBQ-R
1	–	0.07	0.01	0.11	–	–
2	–	0.07	0.01	–	0.1	0.07
3	–	0.11	0.02	–	0.11	0.11
**Study 2**	*OXTR*m	Auditory MSE_AUC_	Visual MSE_AUC_	Verbal behavior	Visual behavior	
	–	0.06	0.02	0.06	0.03	

Composite reliability coefficients reflecting internal consistency and reliability and *R*^2^ coefficients reflecting explanatory power for each construct and model. *Abbreviations*: *OXTR*m *OXTR* DNA methylation, *MSE*_AUC_ area under the multiscale entropy curve, *SD*_CONT_ standard deviation of the continuous time series, *IBQ-R* Revised Infant Behavioral Questionnaire

The oxytocinergic system, social behavior, and their related brain systems have all been shown to be sexually dimorphic [25]. We therefore tested for sex effects in our models by examining differences in path coefficients across male and females using multi-group analysis with pooled standard error in WarpPLS.

##### Sample size

After preprocessing and outlier removal, the final sample consisted of 55 participants with complete epigenetic, neural, and behavioral data. To determine that we had sufficient power for our models with this sample size, we followed the recommendation of Chin and Newsted [83] and computed a power analysis based on the portion of the model with the largest number of predictors. In our models, IBQ-R constructs have the largest number of predictors—up to 3. An extensive literature review suggests a moderate association (*r* = .3 to .5) between measures of neural variability and behavioral outcomes [8, 9, 11]. A multiple regression power analysis [84] determined that 56 participants are needed to detect an effect size (*ρ*^2^) of 0.25 with 3 predictors, 95% power, and *α* = .05.

### Study 2

#### Participants

Sixty-five (31 female) infants were recruited from the greater Charlottesville area to provide a saliva sample, undergo EEG and eye tracking, and receive parent-reported behavioral ratings at 4 months of age (*M* = 131.92 ± 11.59 days). The primary caregiver accompanied the infant to all appointments and provided written informed consent for a protocol approved by the University of Virginia Health and Human Sciences Institutional Review Board. Families were paid $50 for their participation. Eleven infants (4 female) returned within 1 week (*M* = 5.23 ± 2.00 days) to assess the test-retest reliability of brain signal entropy in infancy.

The target sample size for study 2 was determined via a power analysis using effect sizes established in study 1 and following recommendations of Chin and Newsted [83]. In our study 2 model, behavioral constructs have the largest number of predictors—3. A two-tailed multiple regression power analysis [84] determined that 58 participants are needed to detect an effect size of 0.29 with 3 predictors, 95% power, and *α* = .05.

The target sample size for the test-retest reliability analysis was determined via power analysis tables provided by Bujang and Baharum [85] which specify that 10 subjects are sufficient to detect an interclass correlation coefficient (*ICC*) of .70 based on two observations with 80% power.

#### DNA collection and epigenetic analysis

Passive drool collection, DNA isolation, and epigenetic analysis were performed exactly as described in study 1. On average, replicates deviated from the mean ± 2.70%. Methylation levels averaged 42.02% (*SD* = 4.53).

#### EEG acquisition and preprocessing

**Fig. 3:**
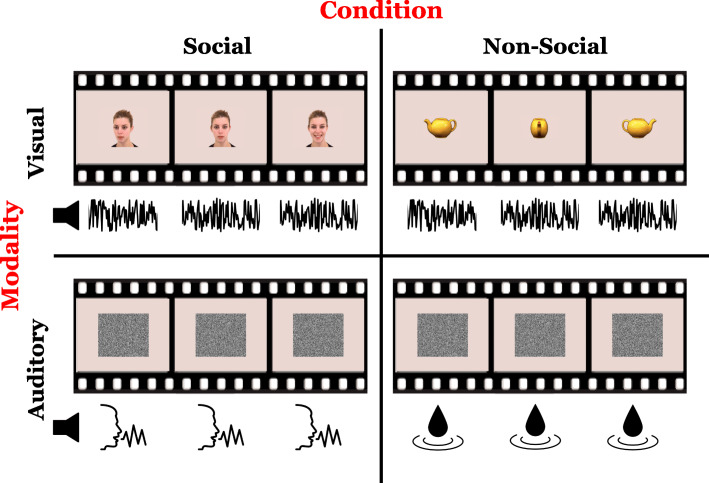
Example stimuli from the study 2 EEG paradigm. The EEG paradigm had a 2 × 2 design with the factors context (social or non-social) and modality (visual or auditory). Visual social stimuli consisted of videos of women turning their heads and smiling. Visual non-social stimuli consisted of videos of common objects rotating. During visual perception, white noise was played in the auditory modality. Auditory social stimuli consisted of infant-directed speech. Auditory non-social stimuli consisted of recordings of water sounds. During auditory perception, a video of static-like salt and pepper noise was played in the visual modality

Infants participated in an EEG paradigm (Fig. 3) consisting of four conditions, resulting in a 2 × 2 design with the factors context (social or non-social) and modality (visual or auditory). Visual social stimuli were obtained from the Amsterdam Dynamic Facial Expression Set [86] and consisted of six female actors each turning towards or away from the camera to the left or right and then smiling for a total of 24 videos. Visual non-social stimuli consisted of dynamic color videos of common objects (e.g., vegetables, toys) rotating. Stimuli were obtained from the Amsterdam Library of Object Images [87], a database of objects photographed in multiple viewing directions. Objects were first cropped and placed on a background matching the social stimuli, then selected such that the non-social stimulus set were matched to the social stimulus set on luminance (*M*_*social*_ *=* 177.16, *M*_*non-social*_ = 178.24, *t* = 0.13, *p* = 0.898, contrast (*M*_*social*_ *=* 52.95, *M*_*non-social*_ = 53.73, *t* = 0.16, *p* = 0.875), and spatial frequency (*M*_*social*_ *=* 13,743.58, *M*_*non-social*_ = 12,067.46, *t* = − 1.92, *p* = 0.068) using custom MATLAB scripts adopted from the SHINE [88] toolbox. The final set consisted of 12 unique objects, each rotating to the left and to the right for a total of 24 videos. Each visual trial had a total duration of 18 s and consisted of six unique 2400-ms videos presented at a visual angle of 8° in a randomized order and with a randomized inter-stimulus interval ranging from 500 to 1000 ms. White noise generated in MATLAB was presented as auditory stimuli during visual trials.

Auditory non-social stimuli consisted of sounds of water from nature (e.g., rain, surf) and household products (e.g., bubbling, splashing) downloaded from http://www.findsounds.com. Auditory social stimuli consisted of naturalistic infant-directed speech recorded from seven English-speaking mothers as they spoke to their preverbal children in their homes [89] downloaded from the Child Language Data Exchange System [90]. Clips containing single-word utterances (e.g., “shoes,” “open,” “hot”) or short phrases (e.g., “oh my goodness,” “uh oh,” “bye-bye”) were extracted from the recordings. Clips containing incoherent speech or background noises were discarded, and individual clips were selected such that the social stimulus set matched the non-social stimulus set on mean fundamental frequency (*M*_*social*_ *=* 311.16, *M*_*non-social*_ = 335.29, *t* = − 1.29, *p* = .200), standard deviation of fundamental frequency (*M*_*social*_ *=* 66.85, *M*_*non-social*_ = 57.82, *t* = 0.95, *p* = .343), and duration (*M*_*social*_ *=* 0.87, *M*_*non-social*_ = 0.97, *t* = − 1.35, *p* = .179) using Praat v6.0.36 [91] and custom MATLAB scripts. The final auditory stimulus set consisted of 60 unique social and 60 unique non-social auditory clips. Clips were grouped by condition (social/non-social) into six 10-clip, 18-s trials such that no word or water sound type repeated within a trial. The inter-stimulus interval between clips ranged from 500 to 1000 ms, randomized across participants. The order of clips within a trial and the presentation order of trials was randomized across participants. Static-like salt and pepper noise videos generated in MATLAB were presented as visual stimuli during auditory blocks.

Stimuli were presented with PsychToolBox v3.0.14 [92] in MATLAB. Infants were seated on their caregiver’s lap approximately 100 cm from a computer monitor throughout the experiment. Caregivers were instructed not to talk or interact with the infant during the course of the experiment. Trials within a block were pseudo-randomized such that visual and auditory trials alternated. A contracting and expanding colorful shape paired with an attention-getting sound was presented within the inter-trial interval to regain the infant’s attention to the center of the computer screen, at which point the experimenter initiated the beginning of the next block. The experimenter viewed the infant via live stream from the control area and could pause the experiment between trials to regain the infant’s attention or compliance if necessary. The EEG session ended when the infant became fussy or inattentive or after 24 blocks. On average, infants completed 7.03 (*SD* = 3.00) blocks of the paradigm.

EEG was recorded from 32 Ag/AgCl active actiCAP slim electrodes (Brain Products GmbH, Germany) affixed to an elastic cap using the 10–20 electrode placement system. The horizontal electrooculogram (EOG) was recorded from two electrodes (F7, F8), which are part of the cap located at the outer canthi of both eyes. The vertical EOG was recorded from two electrodes (FP1, FP2), which are part of the cap on the supraorbital ridge of both eyes. The infant’s head circumference was first obtained to determine the correct cap size prior to capping and gel application. Impedances were assessed via the actiCAP Control Box prior to recording. EEG was amplified with a BrainAmp DC Amplifier and recorded using BrainVision Recorder software with a sampling rate of 5000 Hz, online referenced to FCz, and online band-pass filtered between 0.1 and 1000 Hz. Data were downsampled to 500 Hz prior to subsequent analysis.

EEG preprocessing was performed as in study 1. Visual stimuli were segmented into stimulus-evoked epochs 100 ms pre-stimulus onset to 1000 ms post-stimulus onset with pre-baseline correction. Auditory stimuli were segmented into stimulus-evoked epochs 100 ms pre-stimulus onset to 500 ms post-stimulus onset with pre-baseline correction. Artifact rejection criteria were identical to those in study 1. Participants with at least 20 artifact-free visual segments and 40 artifact-free auditory segments were retained in the analyses. Four participants (6.15%) were excluded for an insufficient number of artifact-free segments, and EEG data failed to save due to a technical error for one additional infant in study 2.

We then completed ICA as in study 1 to remove artifactual components. On average, 3.48 (range 2 to 8) components were removed. The number of components removed did not correlate with MSE within the visual (*r* = .13, *p* = .330) or auditory (*r* = − .15, *p* = .249) modality and did not show significant ERP effects. To include an equivalent number of data points for each condition in the MSE computation, we selected the 20 visual segments (10 social) and the 40 auditory segments (20 social) with a total GFP [8] closest to the median GFP for each participant for inclusion in downstream analyses (5000 data points per condition).

#### Brain signal variability analyses

MSE was calculated exactly as described in study 1. We consider MSE_*AUC*_ for each of the 32 electrodes for scales 1 to 50 (corresponding to 500 to 10 Hz).

Test-retest reliability was assessed for average MSE_*AUC*_ across all conditions via interclass correlation coefficient (*ICC*). *ICC* estimates were calculated using the irr v0.84.1 [93] statistical package in R based on a single rating (*k* = 1), absolute-agreement, 2-way mixed-effects model [94]. EEG data for one infant were excluded due to an insufficient number of artifact-free segments at re-test.

#### Infant behavior

To separately consider how auditory- and visually evoked MSE impacts domain-specific social behaviors, we considered infant social vocal and visual behaviors independently. Infant vocalization behavior was assessed via the 7-item vocalization subscale of the IBQ-RS [43] in which the parent rates how frequently the infant vocalizes during specific activities on a scale ranging from 1 (never) to 7 (always). IBQ-RS data were unavailable for 4 infants. These missing data were imputed via arithmetic mean.

To assess infant visual attention to social information, infants participated in an eye-tracking paradigm in which they viewed 16-s videos of children playing and engaging in nonverbal communication in a naturalistic environment. Stimuli were provided at the courtesy of researchers from the Center for Autism Research at the Children’s Hospital of Philadelphia and included 16 silent video clips of 8 sibling pairs of school-aged children playing with various toys (see [95] for stimulus acquisition details). Videos were presented to the infants in a randomized order. Between each video, infants’ attention was re-oriented to the center of the screen with a colorful spinning object and was paired with an attention-getting sound. Once the infant fixated on the attention-getter for 500 ms, the next video began.

Stimuli were presented using PsychToolBox v3.0.14 [92] for MATLAB. Eye tracking was recorded with Tobii Pro SDK v1.6 for MATLAB and a Tobii X60 eye tracker mounted to a 17-in. computer screen. The infant was seated on the caregiver’s lap throughout the protocol. The caregiver wore darkened glasses to ensure the eye tracker registered only the infant’s pupils and was instructed not to talk or interact with the infant during the course of the experiment. The infant was first positioned 60 cm from the eye tracker and screen. Then, the infant underwent a 5-point calibration procedure in which a colorful, dynamic object expanded and contracted and was paired with an attention-getting sound. If any points failed to calibrate, the calibration procedure was repeated up to two times for those points at which point the eye-tracking paradigm commenced.

Gaze data preprocessing was carried out using custom MATLAB scripts following Tobii recommendations [96], including gap fill-in using linear interpolation for gaps up to 75 ms, average eye computation from binocular data, and median filtering with a length of 7 points (100 ms) [97]. Dynamic areas of interest (AOIs) were drawn around the faces and provided by researchers at the Center for Autism Research at the Children’s Hospital of Philadelphia (see [95] for details).

Social visual attention was defined as the Proportion of Total Fixation Duration to Faces relative to Total Fixation Duration to the entire screen. Calculating a proportional fixation duration is a common technique in infant eye tracking as it accounts for individual differences in overall looking behavior and does not require the implementation of an exclusionary gaze time threshold that would reduce sample size and may produce selection biases. We also calculated the Average Time to First Fixation to Faces to assess how quickly social information captured infant’s attention and Total Fixation Count to Faces to assess the extent of visual exploration of social information. On average, infants completed 8.06 (*SD* = 4.12) blocks of the paradigm. Eye-tracking data was not available for 8 infants due to a failure of the eye tracker to register the infant’s eyes or technical error. These missing data were imputed via arithmetic mean.

#### Experimental design and statistical analysis

We employed PLS-PM using WarpPLS as described in study 1. Methylation values generated in each of the 3 replicate analyses served as indicators for the epigene (*OXTR* methylation) latent variable, Social–Non-social MSE_*AUC*_ difference scores for each of the 32 electrodes in the auditory and visual modalities served as indicators for the brain (Auditory MSE_*AUC*_, Visual MSE_*AUC*_) latent variables, scores from each of the 7 items that constitute the IBQ-RS vocalization subscore served as indicators for the Verbal Behavior latent variable, and the three metrics of social visual attention from the eye-tracking paradigm (Average Time to First Fixation to Faces, Total Fixation Count to Faces, and Proportion of Total Fixation Duration to Faces) served as indicators for the Visual Behavior latent variable.

Outliers were defined as in study 1. The methylation value for one subject was identified as an outlier in a single replicate. This outlier replicate was removed and imputed with the mean of the other two replicate values for this subject. Next, we checked for convergent and discriminant validity by identifying and removing any items that did not significantly load onto its construct or that loaded higher onto another construct for each model. These included T8, TP10, and T9 for Auditory MSE_*AUC*_, F8 and T8 for Visual MSE_*AUC*_, and Average Time to First Fixation for Visual Behavior. Finally, we determined that the square root of the average variance extracted (AVE) was greater than the correlations between constructs. We report construct internal consistency and reliability as indexed through the composite reliability coefficient (recommended value > .60 [80]), and explanatory power through *R*^2^ values in Table 4 for this model.

## Results

### Tissue comparison study

#### Saliva is a reliable tissue for assaying *OXTR* methylation

**Fig. 4:**
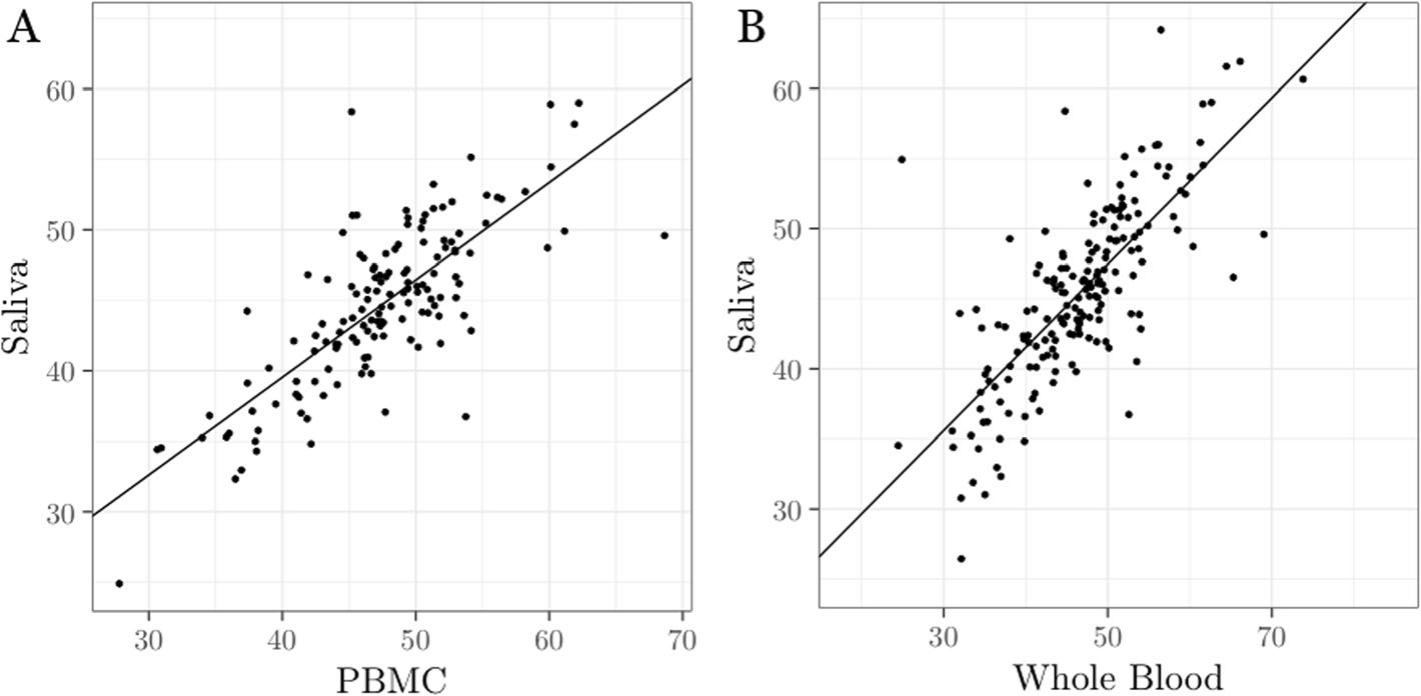
Saliva is a reliable tissue for assaying *OXTR* methylation. DNA methylation values at *OXTR* cytosine-phosphate-guanine (CpG) site -934 are significantly correlated between (**a**) saliva and peripheral blood mononuclear cells (PBMC) (*n* = 142, *r*(140) = .78, *p* < .001), and (**b**) saliva and whole blood (*n* = 182, *r*(180) = .75, *p* < .001)

Previous work has shown that *OXTR* methylation assayed from peripheral blood at CpG site -934 reflects the level of DNA methylation at this site in the brain [46, 50]—the causal tissue for behavior. To expand this marker for use with infants, we first established that *OXTR* methylation levels in saliva, a peripheral tissue more appropriate for vulnerable populations, correspond to *OXTR* methylation levels in blood. Healthy adults provided both passive drool and intravenous blood samples for assessment of whole blood (*n* = 182) and/or PBMC (*n* = 142) methylation. Epigenetic analyses revealed significant correlations between *OXTR* methylation derived from saliva and whole blood (*r*(180) = .75 [95% confidence interval .68, .81], *p* < .001), and saliva and PBMC (*r*(140) = .78, [.70, .83], *p* < .001) at site -934 (Fig. 4).

### Study 1

#### Brain signal entropy is associated with *OXTR* methylation and accounts for individual differences in infant behavior

Brain signal variability can be quantified in different ways. Two of the most commonly examined measures are entropy and SD. This study is the first to directly compare the explanatory power of these measures within one model. In our primary analyses, we consider the area under the MSE curve (MSE_*AUC*_) for scales 1 to 100 (corresponding to 500 to 5 Hz) to obtain a comprehensive picture of the temperodynamic structure of our data, and SD of the continuous time series (SD_*CONT*_) to obtain a measure of the distributional width of the signal.

**Fig. 5:**
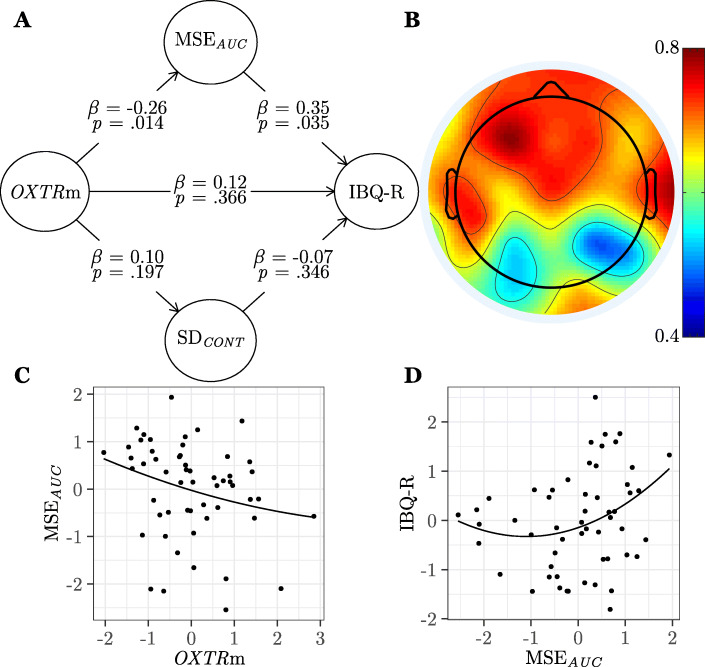
Brain signal entropy is associated with *OXTR* methylation and accounts for individual differences in infant behavior. **a** Results from the partial least squares path model (study 1, model 1, *n* = 55) showing associations between *OXTR* methylation (*OXTR*m), area under the multiscale entropy curve (MSE_*AUC*_) evoked during social perception, standard deviation (SD_*CONT*_) of the continuous time series evoked during social perception, and ratings on the Revised Infant Behavioral Questionnaire (IBQ-R). *β*, path model coefficient; *p*, jackknifed *p* value for coefficient. **b** Topographical map showing loadings of each electrode on the MSE_AUC_ construct. **c** Plot of the significant association between MSE_*AUC*_ and *OXTR*m standardized factor scores. **d** Plot of the significant association between MSE_*AUC*_ and IBQ-R standardized factor scores

We hypothesized that infants with lower *OXTR* methylation (increased gene expression in human cortex [46], presumed increased sensitivity to endogenous oxytocin) would show increased brain signal variability during social perception and would also receive more positive behavioral ratings. To test this hypothesis, we used a multivariate approach to simultaneously model the entire data structure including our epigene (*OXTR* methylation), brain (MSE_*AUC*_, SD_*CONT*_), and behavior (IBQ-R) measures using PLS-PM. The results of this model can be seen in Fig. 5 and Table 4 (model 1). First, we ensured that MSE_*AUC*_ and SD_*CONT*_ were identified as unique, distinguishable constructs in the model by confirming that the loadings for each electrode exceeded .5 [71] for its own construct (MSE_*AUC*_: *M* = .70, SD_*CONT*_: *M* = .67) and that the cross-loadings for each electrode did not exceed .5 onto the other construct (MSE_*AUC*_: *M* < .01, SD_*CONT*_: *M* < .01). We found a significant negative curvilinear association between *OXTR* methylation and MSE_*AUC*_ (*β* = − 0.26, *SE* = 0.12, *p* = .014, *f*^2^ = .07) such that infants with lower *OXTR* methylation showed increased brain signal entropy. We simultaneously found a significant positive curvilinear association between MSE_*AUC*_ and IBQ-R (*β* = 0.35, *SE* = 0.19, *p* = .035, *f*^2^ = .10) such that infants that showed greater entropy during social perception received more positive behavioral ratings. However, we did not find any significant associations between SD_*CONT*_ and *OXTR* methylation (*β* = 0.10, *SE* = 0.12, *p* = .197) or IBQ-R (*β* = − .07, *SE* = 0.17, *p* = .346). While all electrodes loaded significantly onto the MSE_*AUC*_ construct in our model, we obtained significantly higher loading coefficients (*t* = 3.12, *p* = .006)—indicating strongest associations in the model—for frontal and temporal (FP1, FP2, F9, F7, F3, FZ, F4, F8, F10, FC5, FC6, T7, T8, TP9, TP10) electrodes (*M* = .74) compared to all other (C3, CZ, C4, CP5, CP6, P7, P3, PZ, P4, P8, O1, O2) electrodes (*M* = .65). A multi-group analysis revealed no significant differences in path coefficients across male and female participants (all two-tailed *p* values ≥ .753). Table 5 contains results for model 1 using alternate MSE and SD computation methods.

**Table 5:** Model 1 results using alternative brain signal variability computation methods

Model 1: Path coefficients and standard errors				
		From:		
	To:	*OXTR*m	MSE	SD
MSE_*AUC*_ and SD_*CONT*_	MSE	− 0.26 (0.12)*	–	–
	SD	0.10 (0.12)	–	–
	IBQ-R	0.12 (0.34)	0.35 (0.19)*	− 0.07 (0.17)
MSE_*AUC*_ and SD_*TXT*_	MSE	− 0.26 (0.12)*	–	–
	SD	0.10 (0.12)	–	–
	IBQ-R	0.12 (0.34)	0.35 (0.20)*	− 0.06 (0.17)
MSE_*AUC*_ and SD_*AUC*_	MSE	− 0.26 (0.12)*	–	–
	SD	0.15 (0.12)	–	–
	IBQ-R	0.12 (0.35)	0.37 (0.21)*	− 0.08 (0.22)
MSE_*1*_ and SD_*1*_	MSE	− 0.28 (0.13)*	–	–
	SD	0.10 (0.12)	–	–
	IBQ-R	0.09 (0.27)	0.13 (0.59)	− 0.03 (0.17)
MSE_*50*_ and SD_*50*_	MSE	− 0.24 (0.12)*	–	–
	SD	*0.15 (0.12)*^*+*^	–	–
	IBQ-R	0.12 (0.36)	0.36 (0.18)*	− 0.02 (0.18)
MSE_*100*_ and SD_*100*_	MSE	*− 0.20 (0.13)*^*+*^	–	–
	SD	*0.19 (0.11)*^*+*^	–	–
	IBQ-R	0.09 (0.26)	0.18 (0.22)	0.07 (0.37)
MSE_*SDRes*_ and SD_*CONT*_	MSE	− 0.20 (0.12)*	–	–
	SD	0.10 (0.12)	–	–
	IBQ-R	0.09 (0.21)	0.34 (0.12)*	0.06 (0.12)

Path coefficients and (standard errors) are reported for iterations of study 1, model 1 using alternative computation methods for multiscale entropy and standard deviation of the time series. *P* values are estimated with delete-1 jackknifing. Boldfaced (*) effects are significant at the *p* ***≤*** .05 level. Italicized (^+^) effects approach significance at the *p* < .10 level. *Abbreviations*: *MSE*_*AUC*_ area under the multiscale entropy curve, *SD*_*CONT*_ standard deviation of the continuous time series, *SD*_*TXT*_ standard deviation across trials, *SD*_*AUC*_ area under the coarse-grained standard deviation curve, *MSE*_*1*_ multiscale entropy of scale 1, *SD*_*1*_ standard deviation of scale 1, *MSE*_*50*_ multiscale entropy of scale 50, *SD*_*50*_ standard deviation of scale 50, *MSE*_*100*_ multiscale entropy of scale 100, *SD*_*100*_ standard deviation of scale 100, *MSE*_*SDRes*_ multiscale entropy with standard deviation residualized

#### Evoked entropy during social perception is associated with infant social but not non-social behavior

**Fig. 6:**
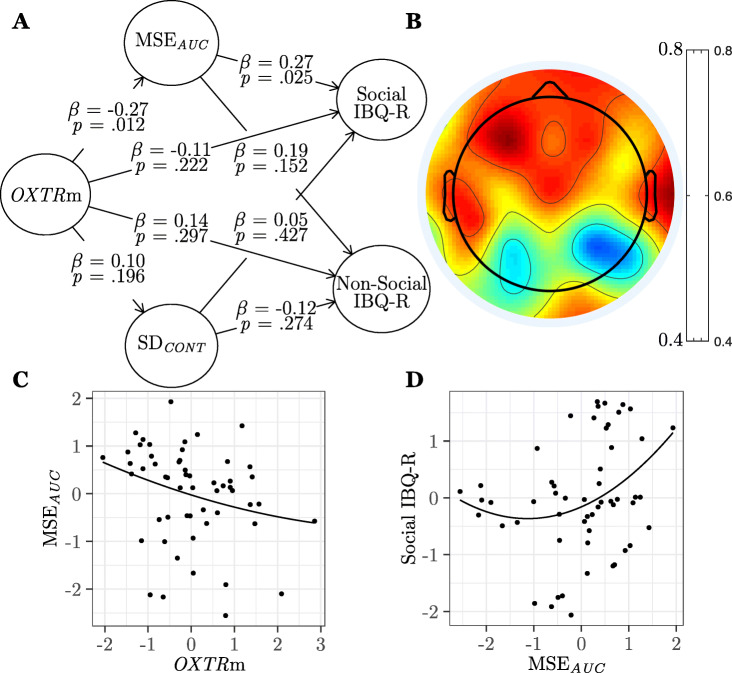
Evoked entropy during social perception is associated with infant social but not non-social behavior. **a** Results from the partial least squares path model (study 1, model 2, *n* = 55) showing associations between *OXTR* methylation (*OXTR*m), area under the multiscale entropy curve (MSE_*AUC*_) evoked during social perception, standard deviation (SD_*CONT*_) of the continuous time series evoked during social perception, and ratings on the Social and Non-Social constructs of the Revised Infant Behavioral Questionnaire (IBQ-R). *β*, path model coefficient; *p*, jackknifed *p* value for coefficient. **b** Topographical map showing loadings of each electrode on the MSE_*AUC*_ construct. **c** Plot of the significant association between MSE_*AUC*_ and *OXTR*m standardized factor scores. **d** Plot of the significant association between MSE_*AUC*_ and Social IBQ-R standardized factor scores

Next, we tested the hypothesis that brain signal variability evoked during social perception would specifically account for individual differences in social, but not non-social, behaviors. To test this hypothesis, we classified items in the IBQ-R subscores into Social and Non-Social constructs (see Table 3). The results of this model can be seen in Fig. 6 and Table 4 (model 2). We found the significant negative curvilinear association between *OXTR* methylation and MSE_*AUC*_ persisted (*β* = − 0.27, *SE* = 0.17, *p* = .012, *f*^2^ = .07), and no significant associations emerged for SD_*CONT*_ (*p*s ≥ .196). As hypothesized, we found that the significant positive curvilinear association between MSE_*AUC*_ and behavior persisted only for the Social IBQ-R construct (*β* = 0.27, *SE* = 0.13, *p* = .025, *f*^2^ = .08). The association between MSE_*AUC*_ and the Non-Social IBQ-R construct was not significant (*β* = 0.19, *SE* = 0.18, *p* = .152). Significant social behavioral indicators suggest that infants with lower *OXTR* methylation and higher MSE_*AUC*_ evoked during social perception vocalize, enjoy cuddling, and approach social situations more, show less fear in social situations, and soothe easier through social interaction. A multi-group analysis revealed no significant differences in path coefficients across male and female participants (all two-tailed *p* values ≥ .320). Table 6 contains results for model 2 using alternate MSE and SD computation methods.

**Table 6:** Model 2 results using alternative brain signal variability computation methods

Model 2: Path coefficients and standard errors				
		From:		
	To:	*OXTR*m	MSE	SD
MSE_*AUC*_ and SD_*CONT*_	MSE	− 0.27 (0.12)*	–	–
	SD	0.10 (0.12)	–	–
	Social IBQ-R	− 0.11 (0.14)	0.27 (0.13)*	0.05 (0.27)
	Non-social IBQ-R	0.14 (0.27)	0.19 (0.18)	− 0.12 (0.20)
MSE_*AUC*_ and SD_*TXT*_	MSE	− 0.27 (0.12)*	–	–
	SD	0.10 (0.12)	–	–
	Social IBQ-R	− 0.04 (0.17)	0.23 (0.11)*	0.10 (0.16)
	Non-social IBQ-R	0.04 (0.49)	0.22 (0.24)	0.03 (0.29)
MSE_*AUC*_ and SD_*AUC*_	MSE	− 0.27 (0.12)*	–	–
	SD	*0.15 (0.12)*^*+*^	–	–
	Social IBQ-R	− 0.11 (0.14)	0.29 (0.14)*	0.00 (0.13)
	Non-social IBQ-R	0.14 (0.27)	0.13 (0.20)	− 0.19 (0.21)
MSE_*1*_ and SD_*1*_	MSE	− 0.28 (0.13)*	–	–
	SD	0.10 (0.12)	–	–
	Social IBQ-R	− 0.20 (0.17)	0.11 (0.34)	− 0.21 (0.26)
	Non-social IBQ-R	0.14 (0.36)	0.10 (0.13)	− 0.22 (0.15)
MSE_*50*_ and SD_*50*_	MSE	− 0.25 (0.12)*	–	–
	SD	*0.15 (0.12)*^*+*^	–	–
	Social IBQ-R	− 0.10 (0.15)	0.31 (0.13)*	0.03 (0.14)
	Non-social IBQ-R	0.15 (0.38)	0.08 (0.23)	− 0.23 (0.22)
MSE_*100*_ and SD_*100*_	MSE	*− 0.20 (0.13)*^*+*^	–	–
	SD	*0.19 (0.12)*^*+*^	–	–
	Social IBQ-R	− 0.12 (0.17)	0.21 (0.20)	− 0.04 (0.30)
	Non-social IBQ-R	0.14 (0.38)	− 0.08 (0.32)	− 0.25 (0.17)
MSE_*SDRes*_ and SD_*CONT*_	MSE	− 0.21 (0.12)*	–	–
	SD	0.10 (0.12)	–	–
	Social IBQ-R	− 0.14 (0.15)	0.30 (0.14)*	0.09 (0.76)
	Non-social IBQ-R	0.16 (0.32)	0.20 (0.20)	− 0.12 (0.75)

Path coefficients and (standard errors) are reported for iterations of study 1, model 2 using alternative computation methods for multiscale entropy and standard deviation of the time series. *P* values are estimated with delete-1 jackknifing. Boldfaced (*) effects are significant at the *p* ***≤*** .05 level. Italicized (^+^) effects approach significance at the *p* < .10 level. *Abbreviations*: *MSE*_*AUC*_ area under the multiscale entropy curve, *SD*_*CONT*_ standard deviation of the continuous time series, *SD*_*TXT*_ standard deviation across trials, *SD*_*AUC*_ area under the coarse-grained standard deviation curve, *MSE*_*1*_ multiscale entropy of scale 1, *SD*_*1*_ standard deviation of scale 1, *MSE*_50_ multiscale entropy of scale 50, *SD*_*50*_ standard deviation of scale 50, *MSE*_*100*_ multiscale entropy of scale 100, *SD*_*100*_ standard deviation of scale 100, *MSE*_SDRes_ multiscale entropy with standard deviation residualized

#### Ongoing entropy does not show social-behavioral specificity

**Fig. 7:**
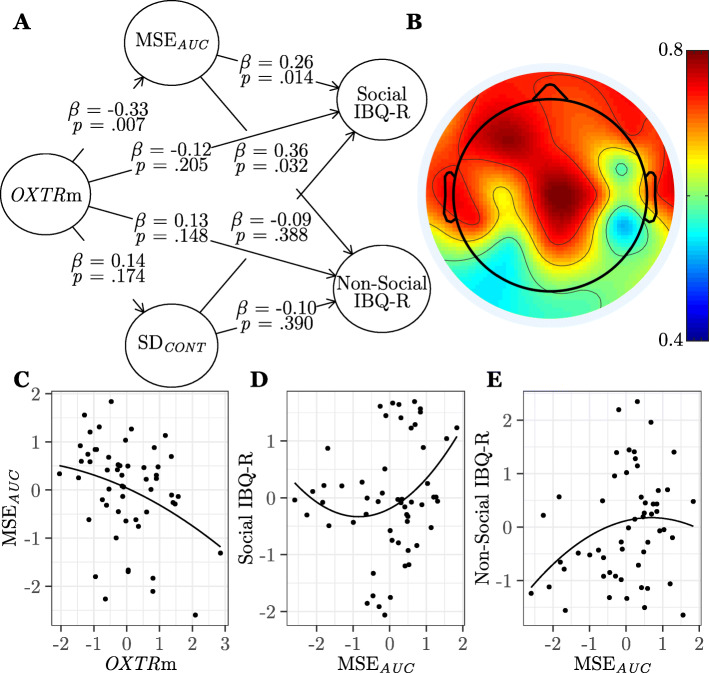
Ongoing entropy does not show social-behavioral specificity. **a** Results from the partial least squares path model (study 1, model 3, *n* = 55) showing associations between *OXTR* methylation (*OXTR*m), ongoing area under the multiscale entropy curve (MSE_*AUC*_), ongoing signal standard deviation of the continuous time series (SD_*CONT*_), and ratings on the Social and Non-Social constructs of the Revised Infant Behavioral Questionnaire (IBQ-R). *β*, path model coefficient; *p*, jackknifed *p*-value for coefficient. **b** Topographical map showing loadings of each electrode on the MSE_*AUC*_ construct. **c** Plot of the significant association between MSE_*AUC*_ and *OXTR*m standardized factor scores. **d** Plot of the significant association between MSE_*AUC*_ and Social IBQ-R standardized factor scores. **e** Plot of the significant association between MSE_*AUC*_ and Non-Social IBQ-R standardized factor scores

Finally, we examined whether these associations between *OXTR* methylation, brain signal variability, and infant social behavior occurred specifically due to the fact that infants were engaged in social perception during brain signal measurement, or if spontaneous, ongoing brain signal variability is associated with infant behavior regardless of perceptual context. To assess ongoing neural variability, we randomly extracted segments of brain signal from the inter-stimulus interval that were not time-locked to and did not overlap with stimulus presentation and re-ran the previous model with brain signal variability calculated on this spontaneous, ongoing signal. We found evoked and ongoing MSE_*AUC*_ are significantly correlated across all electrodes (*r*s range from .57 to .84, all *p*s < .001). We again found that the significant negative curvilinear association between *OXTR* methylation and MSE_*AUC*_ persisted (*β* = − 0.33, *SE* = 0.13, *p* = .007, *f*^2^ = .11), whereas no significant associations emerged for SD_*CONT*_ (*p*s ≥ .174). Interestingly, this analysis revealed a significant positive curvilinear association between MSE_*AUC*_ and both Social (*β* = 0.26, *SE* = 0.12, *p* = .014, *f*^2^ = .08) and Non-Social (*β* = 0.36, *SE* = 0.19, *p* = .032, *f*^2^ = .11) IBQ-R constructs, suggesting spontaneous, ongoing entropy, outside of a perceptual context, is associated with general, non-context-specific infant behavior. Significant behavioral indicators demonstrate that infants who showed greater ongoing brain signal entropy soothe easier through both social and non-social means, are more likely to approach and show excitement for both social and non-social activities, show greater perceptual sensitivity to non-social stimuli, enjoy cuddling more, and show less fear in social situations. The results of this model can be seen in Fig. 7 and Table 4 (model 3). A multi-group analysis revealed no significant differences in path coefficients across male and female participants (all two-tailed *p* values ≥ .316). Table 7 contains results for model 3 using alternate MSE and SD computation methods.

**Table 7:** Model 3 results using alternative brain signal variability computation methods

Model 3: Path coefficients and standard errors				
		From:		
	To:	*OXTR*m	MSE	SD
MSE_*AUC*_ and SD_*CONT*_	MSE	− 0.33 (0.13)*	–	–
	SD	0.14 (0.15)	–	–
	Social IBQ-R	− 0.12 (0.15)	0.26 (0.12)*	− 0.09 (0.31)
	Non-social IBQ-R	0.13 (0.12)	0.36 (0.19)*	− 0.10 (0.37)
MSE_*AUC*_ and SD_TXT_	MSE	− 0.33 (0.13)*	–	–
	SD	0.14 (0.15)	–	–
	Social IBQ-R	− 0.04 (0.16)	0.25 (0.13)*	0.12 (0.14)
	Non-social IBQ-R	− 0.02 (0.14)	0.45 (0.25)*	− 0.16 (0.80)
MSE_*AUC*_ and SD_*AUC*_	MSE	− 0.33 (0.13)*	–	–
	SD	*0.19 (0.15)*^*+*^	–	–
	Social IBQ-R	− 0.12 (0.15)	0.26 (0.13)*	0.03 (0.29)
	Non-social IBQ-R	0.13 (0.12)	0.35 (0.23)	− 0.09 (0.55)
MSE_*1*_ and SD_*1*_	MSE	− 0.27 (0.14)*	–	–
	SD	0.14 (0.15)	–	–
	Social IBQ-R	− 0.21 (0.18)	− 0.17 (0.68)	− 0.22 (0.36)
	Non-social IBQ-R	0.12 (0.31)	0.11 (0.15)	− 0.15 (0.16)
MSE_*50*_ and SD_*50*_	MSE	− 0.33 (0.13)*	–	–
	SD	0.19 (0.15)	–	–
	Social IBQ-R	− 0.13 (0.15)	0.24 (0.13)*	− 0.08 (0.26)
	Non-social IBQ-R	0.10 (0.14)	0.27 (0.21)	− 0.01 (0.23)
MSE_*100*_ and SD_*100*_	MSE	*− 0.23 (0.14)*^*+*^	–	–
	SD	*0.24 (0.14)*^*+*^	–	–
	Social IBQ-R	− 0.13 (0.16)	0.40 (0.14)*	− 0.07 (0.12)
	Non-social IBQ-R	0.11 (0.22)	0.20 (0.22)	− 0.10 (0.20)
MSE_*SDRes*_ and SD_*CONT*_	MSE	− 0.28 (0.11)*	–	–
	SD	0.14 (0.15)	–	–
	Social IBQ-R	− 0.13 (0.16)	0.28 (0.13)*	*− 0.16 (0.11)*^*+*^
	Non-social IBQ-R	0.17 (0.33)	*0.30 (0.19)*^*+*^	0.04 (0.20)

Path coefficients and (standard errors) are reported for iterations of Study 1, Model 3 using alternative computation methods for multiscale entropy and standard deviation of the time series. *P*-values are estimated with delete-1 jackknifing. Boldfaced (*) effects are significant at the *p* ***≤*** .05 level. Italicized (^+^) effects approach significance at the *p* < .10 level. *Abbreviations*: *MSE*_*AUC*_ area under the multiscale entropy curve, *SD*_*CONT*_ standard deviation of the continuous time series, *SD*_*TXT*_ standard deviation across trials, *SD*_*AUC*_ area under the coarse-grained standard deviation curve, *MSE*_*1*_ multiscale entropy of scale 1, *SD*_*1*_ standard deviation of scale 1, *MSE*_*50*_ multiscale entropy of scale 50, *SD*_*50*_ standard deviation of scale 50, *MSE*_*100*_ multiscale entropy of scale 100, *SD*_*100*_ standard deviation of scale 100, *MSE*_SDRes_ multiscale entropy with standard deviation residualized

### Study 2

#### Entropy shows modality-specific associations with infant social behavior

**Fig. 8:**
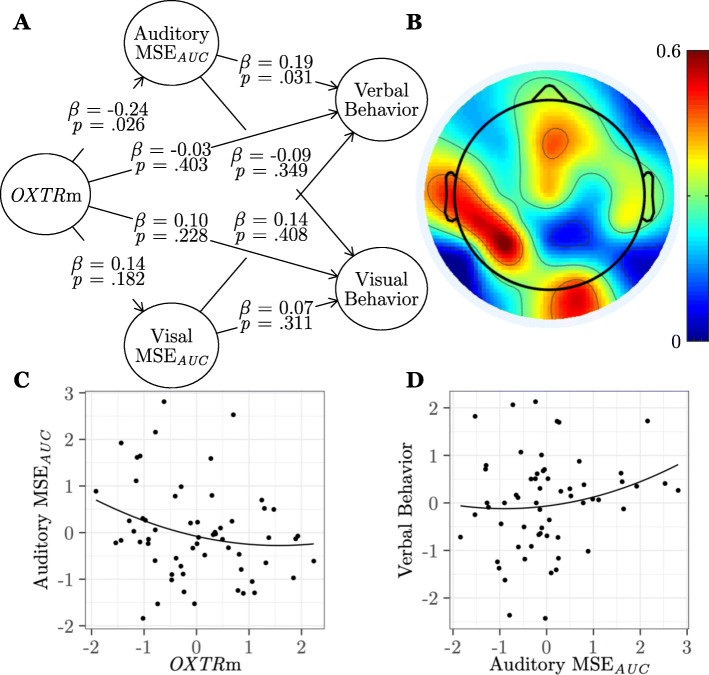
Entropy shows modality-specific associations with infant social behavior. **a** Results from the partial least squares path model (study 2, *n* = 60) showing associations between *OXTR* methylation (*OXTR*m), area under the multiscale entropy curve (MSE_*AUC*_) for auditory and visual modalities, and infant social verbal and visual behavior. *β*, path model coefficient; *p*, jackknifed *p*-value for coefficient. **b** Topographical map showing loadings of each electrode on the Auditory MSE_*AUC*_ construct. **c** Plot of the significant association between Auditory MSE_*AUC*_ and *OXTR*m standardized factor scores. **d** Plot of the significant association between Auditory MSE_*AUC*_ and infant verbal behavior standardized factor scores

In study 2, we consider modality-specific associations between *OXTR* methylation, brain signal entropy evoked by social relative to non-social stimuli, and infant verbal (vocalization) and visual (attention to faces) social behavior in 4-month-old infants. We find, as in study 1, a negative association between *OXTR* methylation and social auditory-evoked MSE_*AUC*_ (*β* = − 0.25, *SE* = 0.12, *p* = .026, *f*^2^ = .06) and a positive association between social auditory-evoked MSE_*AUC*_ and infant social (verbal) behavior (*β* = 0.19, *SE* = 0.10, *p* = .031, *f*^2^ = .04). However, social auditory-evoked MSE_*AUC*_ is not associated with infant social visual behavior (*β* = − 0.09, *p* = .349). There is also no association between *OXTR* methylation and visually evoked MSE_*AUC*_ (*β* = 0.14, *p* = .182), nor between visually evoked MSE_*AUC*_ and visual (*β* = 0.07, *p* = .311) or verbal (*β* = 0.14, *p* = .408) social behavior at 4 months of age. Topographical loadings for auditory-evoked MSE_*AUC*_ load strongest onto left temporal electrodes. The results of this model can be seen in Fig. 8 and Table 4 (study 2).

#### Multiscale entropy is a reliable measure in infancy

**Fig. 9:**
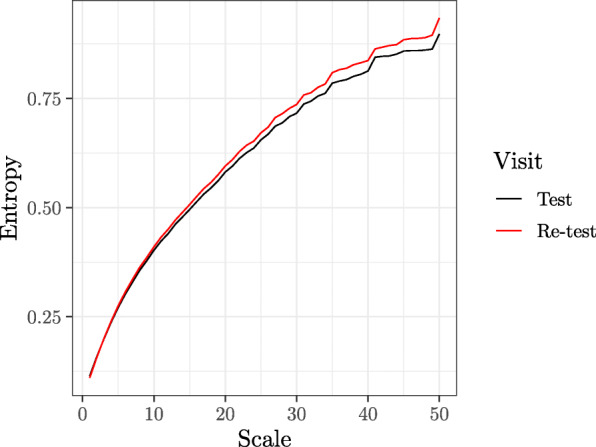
Multiscale entropy is a reliable measure in infancy. Average multiscale entropy curves for scales 1 to 50 (500 to 10 Hz) are plotted for ten study 2 infants who underwent EEG at 4 months of age (test visit, black), and repeated the procedure within 1 week (re-test visit, red). We find good reliability (*ICC* = .73, *p* = .004) across the 1-week timespan

Finally, a subset of 4-month-old infants returned within 1 week of their study appointment to determine the test-retest reliability of brain signal entropy (*n =* 10). MSE was found to show good reliability within 1 week (*ICC* = .73, *p* = .004). MSE test-retest reliability curves are plotted in Fig. 9.

## Discussion

Using a multivariate, prediction-based model, we show for the first time associations between early-life *OXTR* methylation, brain signal entropy, and infant behavior across two independent studies. Specifically, infants with lower levels of *OXTR* methylation (and likely increased sensitivity to endogenous oxytocin) show increased MSE during social perception, which is associated with more positive ratings specific to social behaviors. Our study 1 results demonstrate that these associations are (1) measure-specific—entropy, but not signal variance, links *OXTR* methylation and infant behavior, and (2) context-sensitive—entropy evoked during social perception specifically explains social behavior only. Our study 2 results replicate the association between *OXTR* methylation, social auditory-evoked MSE, and infant social behavior and suggest a modality-specific effect in infancy. Specifically, at 4 months of age, we only obtained significant epigene-brain-behavior associations within the auditory modality.

The current findings critically extend a growing body of research highlighting MSE as a powerful indicator of behavioral and developmental outcomes [14, 98, 99] and establish the reliability of this measure in infancy for the first time. In our analyses, we compared the explanatory power of two measures of brain signal variability and found that only MSE shows significant links between *OXTR* methylation and infant behavior. SD, a measure of overall distributional width, has been an effective measure for identifying group-level differences between healthy and clinical populations [54–56] or young and old adults [66, 74]. However, we may have found significant results with MSE and not SD in our sample of healthy infants because MSE is sensitive to temporal dependencies in a time series (Fig. 2) and is measured across multiple time scales. These distinctions may enable MSE to index neurodevelopmental changes that occur very early in life or are indicative of individual differences within even the healthy range of the continuum of human social behavior.

In addition to understanding what measures of neural variability are capable of explaining developmental or behavioral differences, it is also important to understand when neural variability is exploited to benefit perception or behavior. Neural activity can be categorized into two primary states: spontaneous ongoing brain activity, considered the default or “resting” state of the brain, and evoked brain activity that occurs in response to specific stimulation. It is thought that spontaneous, ongoing variability predominates in the brain, and evoked variability represents a relatively small proportion of overall variability that operates on top of ongoing variability to enable relevant behaviors [3], perhaps by optimizing sensory encoding and enhancing subsequent representations [10]. Our MSE study 1 results are in line with this understanding of brain variability. Infants that show greater ongoing MSE receive more positive behavioral ratings across contexts, perhaps reflecting a more dynamic, mature neural system [21, 52, 100] in general (trait) among these infants. However, infants that show higher MSE during social perception (state) might show enhanced social behaviors because they are able to build better perceptual and cognitive representations of complex social stimuli specifically, enabling particular sensitivity and flexibility to social stimuli. Spontaneous and evoked activity are understood to be intricately linked [19, 31, 101–103], and indeed, we find evoked and ongoing MSE are significantly correlated. These findings corroborate other research [10] showing that brain signal entropy reflects both trait-like differences across individuals and state-like variability within an individual.

Results of study 2 suggest a modality-specific association between brain signal variability and infant social behavior. Specifically, we find 4-month-old infants that show increased entropy to social, relative to non-social stimuli within the auditory modality vocalize more frequently. However, brain signal entropy evoked during visual perception was not associated with their verbal behavior. Neither entropy within the visual nor the auditory modality was associated with the infant’s visual attention to social stimuli at 4 months of age. These results replicate and extend study 1 by including a younger infant sample, measuring *OXTR* methylation, EEG, and behavior at the same timepoint, and introducing both visual and auditory stimuli across social and non-social domains. Together, these findings highlight the importance of early-life social auditory perception for the developing infant. Converging lines of research suggest that young infants predominately rely on auditory cues for social perception [104]. For example, 5-month-old infants consistently respond differentially to positive and negative voices but not faces [105], suggesting infants are more sensitive to voices than faces in early infancy. Furthermore, in a social referencing paradigm in which infants use the emotional expression of their mother to regulate their own behavior, mother’s vocalized fear alone, but not fearful face alone, towards an object was sufficient for 12-month-old infants to avoid the object [106].

This auditory dominance in infancy is unsurprising. As with many mammals, the auditory system develops much earlier than the visual system [107], and in humans, visual acuity does not reach adult levels until the third year of life [108]. Our modality-specific results support the hypothesis that neural variability is exploited to benefit perception or behavior from early in development. It is possible that young infants show higher brain signal entropy during social auditory perception because of enhanced perceptual experience with that stimulus class, enabling more robust cognitive representations and more effective production of vocalizations.

Our results have important implications for our understanding of neurodevelopmental disorders such as autism. We find the highest loadings for socially evoked MSE over frontal and temporal electrodes (Figs. 5b, 6b, 8b), indicating that irregularity in these regions most accounts for our epigene-brain-behavior associations. These regions are directly implicated in the oxytocinergic signaling pathway [109] and are critical for supporting social-cognitive processes [110] that emerge early in infancy [24]. Individuals with autism—a neurodevelopmental disorder marked by social impairment—show atypical neural development, particularly in frontal and temporal lobes [111]. These differences are thought to be reflected in altered brain signal entropy that occurs in autism [98, 112] even before diagnostic behaviors emerge [59, 99]. Differences within the oxytocinergic system are also implicated in autism, including increased *OXTR* methylation in both the brain and blood at site -934 [46]. Here we present data from two independent infant samples that may provide the foundation for a unifying, mechanistic account of social neurodevelopment, showing that early-life epigenetic differences in *OXTR* are associated with brain signal entropy during social perception to explain individual differences in social behavior in a context- and modality-specific manner.

### Limitations and future directions

Our approach specifically targeted a single epigenetic site because of its association with gene expression in human cortex [46], variability across the general population [47], and sensitivity to early-life experiences in both causal (neural) and peripheral tissue [46, 50]. However, complex social behavioral phenotypes do not arise from the actions of a single molecular system. While it is remarkable that DNA methylation of a single *OXTR* CpG site significantly explains neural MSE, we acknowledge that the effect sizes are moderate and the overall variance explained by our models is fairly low. These results may be strengthened through a more comprehensive examination of markers of individual differences in oxytocinergic system function. Other molecular systems capable of impacting neural variability have been identified in human and animal models, such as the vasopressin [30] and dopaminergic [113] systems. Future longitudinal studies should simultaneously assay brain signal variability while targeting multiple genetic and molecular systems to gain a more complete understanding of the factors impacting neural variability in development.

Critically, we established here that *OXTR* methylation at site -934 is highly conserved in blood and saliva in a secondary, adult population, and we have previously shown that methylation at this site does not vary across cell types [48]. Demonstrating these associations is important because methylation plays a role in cell-type differentiation which may cause methylation patterns to vary across tissues. However, these results warrant replication in a developmental sample because the cellular composition of tissues may also vary with age [114].

Given the association between signal variability, signal detection [2, 3], and sensory encoding [10], we hypothesize that oxytocin ultimately regulates social behavior by increasing perceptual and attentional biases [48] to social information that drive experience-dependent neural development and are reflected in increased brain signal entropy during social perception. One limitation of the present studies is that we primarily relied on a parent-report measure to examine individual differences in infant behavior, although it should be noted that this measure has been validated by showing good correspondence with direct behavioral measures [36–38]. Nonetheless, future studies should include additional overt measures of infant perception and attention to more directly test the proposed attentional mechanism underlying the epigene-brain-behavior associations observed in the current studies.

Here, we add to important work to establish the reliability and validity of MSE [63, 115, 116] and to suggest its validity as a measure of individual differences [116]. However, a limitation to these and the majority of other studies investigating EEG variability metrics to date is the focus on a limited number of manually selected metrics with minimal consideration of alternatives [117]. A comprehensive examination of how recording, preprocessing, and time series algorithm choices influence the ability to detect associations with individual difference factors, be it age, disease state, or behavioral outcomes, is needed.

It is also important to acknowledge that future confirmatory research with larger samples is needed to more directly assess the theorized associations put forth by our models. Nonetheless, our use of predictive modeling capable of accounting for curvilinear associations between variables is a strength of the current studies. Few biological associations are likely linear in nature. Instead, many phenomena show u- or inverted u-shaped functions in which extremes on either end of the spectrum diverge from an optimal level [75–77]. Including high-risk populations to study normative and atypical development concurrently will be critical in future work to identify how neurobiological processes influence pathways of development towards or away from positive outcomes. Furthermore, both the quantity *and* the timing of oxytocin-regulated brain signal entropy likely impacts social development, and too much variability too soon in development, or too little too late, may result in suboptimal outcomes. Future research employing a longitudinal approach will therefore be critical for understanding how one’s genes, brain, and behavior interact throughout development to create a uniquely social individual.

## Conclusions

The first year of life constitutes a time of rapid and dramatic changes in behavioral repertoire, cognitive ability, and neural architecture. During this time, developing infants are confronted with the daunting task of making sense of the world as they are bombarded with competing, fluctuating, and often ambiguous external stimuli. Understanding how the brain develops to form accurate models of the external world and generates appropriate behavioral responses is a significant and critical question of widespread multidisciplinary interest. Social information is particularly complex and dynamic, and the ability to perceive, interpret, elicit, and respond to social information is critical for an infant’s ability to survive and learn [118]. Our results suggest a mechanism by which early-life individual differences in the endogenous oxytocinergic system may drive unique neurodevelopmental trajectories affecting social behavior. These results hold implications for identifying individuals at risk for atypical development before behavioral manifestations of disorder occur and suggest potential biomarkers with probable diagnostic, therapeutic, and prognostic value.

## Supplementary information

**Additional file 1.** Criterion latent variable quartile plots for all significant effects for each model.

## Data Availability

The datasets used and/or analyzed during the current study are available from the corresponding author on reasonable request.

## Abbreviations

AOIs: Areas of interest
AUC: Area under the curve
AVE: Average variance extracted
CBSEM: Covariance-based structural equation modeling
CpG: Cytosine-phosphate-guanine
DNA: Deoxyribonucleic acid
EEG: Electroencephalography
EOG: Electrooculogram
ERP: Event-related potential
fMRI: Functional magnetic resonance imaging
GFP: Global field power
IBQ-R: Revised Infant Behavior Questionnaire
IBQ-RS: Short Revised Infant Behavior Questionnaire
ICA: Independent components analysis
ICC: Interclass correlation coefficient
MSE: Multiscale entropy
MSE_*1*_: Multiscale entropy of scale 1
MSE_*50*_: Multiscale entropy of scale 50
MSE_*100*_: Multiscale entropy of scale 100
MSE_*AUC*_: Area under the multiscale entropy curve
MSE_*SDRes*_: Multiscale entropy with standard deviation residualized
*OXTR*: Oxytocin receptor gene
PBMC: Peripheral blood mononuclear cell
PLS-PM: Partial least squares path modeling
SD: Standard deviation
SD_*1*_: Standard deviation of scale 1
SD_*50*_: Standard deviation of scale 50
SD_*100*_: Standard deviation of scale 100
SD_*AUC*_: Area under the course-grained standard deviation curve
SD_*CONT*_: Standard deviation of the continuous time series
SD_*TXT*_: Standard deviation across trials
SE: Standard error
