# Correction to: Epigenetic tuning of brain signal entropy in emergent human social behavior

**DOI:** 10.1186/s12916-020-01758-9

**Published:** 2020-09-12

**Authors:** Meghan H. Puglia, Kathleen M. Krol, Manuela Missana, Cabell L. Williams, Travis S. Lillard, James P. Morris, Jessica J. Connelly, Tobias Grossmann

**Affiliations:** 1 Department of Psychology, University of Virginia, Charlottesville, VA 22904 USA; 2 Department of Neurology, University of Virginia, P.O. Box 800834, Charlottesville, VA 22908 USA; 3 Max Planck Institute for Human Cognitive and Brain Sciences, 04103 Leipzig, Germany; 4 Department of Early Child Development and Culture, Leipzig University, 04109 Leipzig, Germany

**Correction to: BMC Med 18, 244 (2020)**

**https://doi.org/10.1186/s12916-020-01683-x**

Following the publication of the original article [1], the following errors in Figs. 1 and 6 were brought to our attention:

1. The curves were inadvertently omitted from the graph in Fig. 1.
2. The colour bar in Fig. 6 appeared blank in the published article.

The correct figures are presented here below:

**Figure 1:**
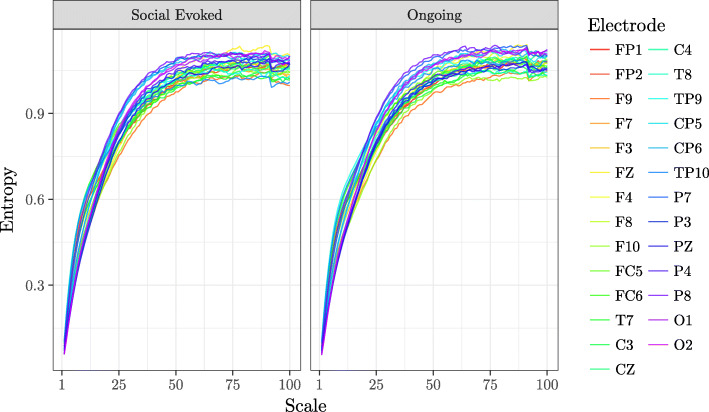
Fig. 1

**Figure 2:**
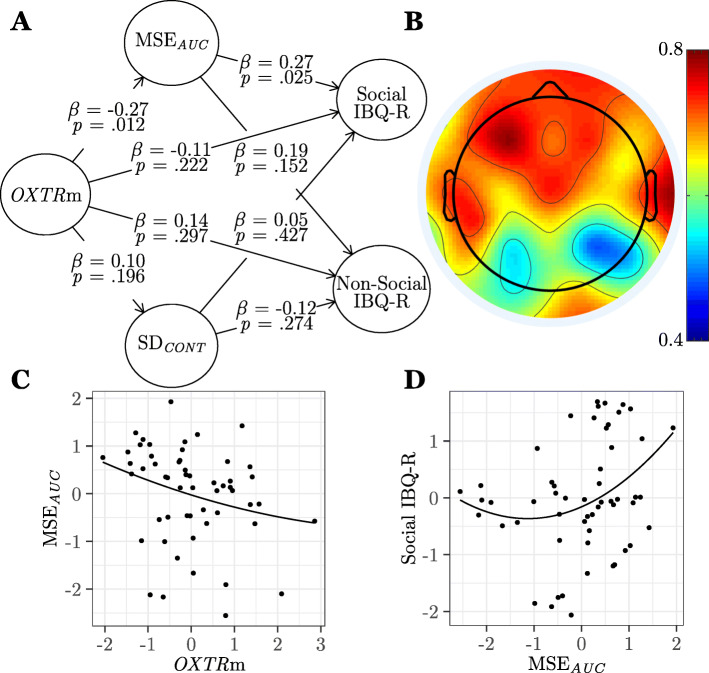
Fig. 6

## References

[1] Puglia MH (2020). Epigenetic tuning of brain signal entropy in emergent human social behavior. BMC Med.

