# Supplementary material for: Epigenetic tuning of brain signal entropy in emergent human social behavior
Source: BMC Med. 2020 Aug 17;18:244. doi: 10.1186/s12916-020-01683-x (PMC7429788; doi:10.1186/s12916-020-01683-x)
Supplement: Supplementary file 1 — Additional file 1. Criterion latent variable quartile plots for all significant effects for each model. [file 12916_2020_1683_MOESM1_ESM.docx]

**Additional File 1: Criterion latent variable quartile plots for all significant effects for each model.** For each significant effect, the dataset is divided into quartiles and the mean value of each criterion latent variable for each quartile is plotted to determine whether the nonlinear functions discovered by the WarpPLS software represent good approximations of the true underlying functions in the data. The shape of these plots can be compared to the best-fitting curves plotted for each model in Figures 5-8.

**Model 1.** Quantile plot of the significant association between evoked MSE*_AUC_* and *OXTR*m (left, compare to Figure 5C), and evoked MSE*_AUC_* and IBQ-R (right, compare to Figure 5D). Abbreviations: MSE*_AUC_*, area under the multiscale entropy curve; *OXTR*m, DNA methylation of the oxytocin receptor gene; IBQ-R, Revised Infant Behavioral Questionnaire.

**Model 2.** Quantile plot of the significant association between socially-evoked MSE*_AUC_* and *OXTR*m (left, compare to Figure 6C), and socially-evoked MSE*_AUC_* and Social IBQ-R (right, compare to Figure 6D). Abbreviations: MSE*_AUC_*, area under the multiscale entropy curve; *OXTR*m, DNA methylation of the oxytocin receptor gene; IBQ-R, Revised Infant Behavioral Questionnaire.

**Model 3.** Quantile plot of the significant association between ongoing MSE*_AUC_* and *OXTR*m (left, compare to Figure 7C), ongoing MSE*_AUC_* and Social IBQ-R (center, compare to Figure 7D), and ongoing MSE*_AUC_* and Non-Social IBQ-R (right, compare to Figure 7E). Abbreviations: MSE*_AUC_*, area under the multiscale entropy curve; *OXTR*m, DNA methylation of the oxytocin receptor gene; IBQ-R, Revised Infant Behavioral Questionnaire.

**Study 2.** Quantile plot of the significant association between Social – Non-Social auditory-evoked MSE*_AUC_* and *OXTR*m (left, compare to Figure 8C), Social – Non-Social auditory-evoked MSE*_AUC_* and Verbal Behavior (right, compare to Figure 8D). Abbreviations: MSE*_AUC_*, area under the multiscale entropy curve; *OXTR*m, DNA methylation of the oxytocin receptor gene.
